# Determination of Mechanical and Fracture Properties of Silicon Single Crystal from Indentation Experiments and Finite Element Modelling

**DOI:** 10.3390/ma14226864

**Published:** 2021-11-14

**Authors:** Petr Skalka, Michal Kotoul

**Affiliations:** 1 Institute of Solid Mechanics, Mechatronics and Biomechanics, Brno University of Technology, Technická 2896/2, 616 69 Brno, Czech Republic; skalka@fme.vutbr.cz; 2 Faculty of Special Technology, Alexander Dubček University of Trenčín in Trenčín, Studentska 2, 911 50 Trenčín, Slovakia

**Keywords:** micro-indentation, mechanical and fracture properties identification, finite element analysis, optimisation analysis

## Abstract

It is well-known that cracks are observed around the impression during indentation of brittle materials. The cracks inception depends on load conditions, material and indenter geometry. The paper aims to use experimental micro-indentation data, FE simulations with cohesive zone modelling, and an optimisation procedure to determine the cohesive energy density of silicon single crystals. While previous studies available in the literature, which use cohesive zone finite element techniques for simulation of indentation cracks in brittle solids, tried to improve methods for the evaluation of material toughness from the indentation load, crack size, hardness, elastic constants, and indenter geometry, this study focuses on the evaluation of the cohesive energy density 2*Γ* from which the material toughness can be easily determined using the well-known Griffith-Irwin formula. There is no need to control the premise of the linear fracture mechanics that the cohesive zone is much shorter than the crack length. Hence, the developed approach is suitable also for short cracks for which the linear fracture mechanics premise is violated.

## 1. Introduction

In recent decades, a number of studies devoted to identification of material properties such as Young’s modulus, yield stress, and work hardening modulus by using experimental indentation data, finite element (FE) simulations, and optimisation procedures for solving inverse problems have occurred in the literature. Various optimisation techniques have been used by researchers, see, e.g., [1,2,3,4] to determine material properties from indentation load–displacement curves tests. Identification of elastic and/or elasto-visco-plastic constitutive laws from indentation tests in terms of general theoretical framework of inverse problems solution has been described in [5,6]. With respect to brittle materials, cohesive zone FE simulations of indentation cracking have been performed e.g., in [7,8,9,10] to investigate the crack morphology, the change of crack length with indenter shape, a quantitative evaluation of the threshold load for indentation fracture, and to explore a limitation of analytical models such as Lawn-Evans-Marshall model [11]. For indentation crack initiation and propagation modelling a cohesive interface consisting of cohesive elements is placed in the plane of potential cracking and only mode I type crack is considered. The behaviour of the cohesive elements in this interface is governed by a traction-separation law which mostly has the bilinear form characterised by three parameters- peak cohesive traction *σ*_max_, corresponding damage-initiating displacement ∆*_c_* and failure displacement ∆*_sep_*. It was shown in [12] that the cohesive energy density and the peak cohesive traction play a far more important role than the shape of the cohesive traction—separation curve in predicting the final fracture behaviour. In case of the bilinear form of the cohesive traction—separation law the cohesive energy density (critical fracture energy) 2*Γ* can be calculated by 2$\Gamma =\frac{1}{2}\sigma_{\max}\Delta_{c}.\;$Critical review of various cohesive zone models is given in [13]. Contrary to analytical approaches, cohesive interface FE simulations exhibit a natural advantage consisting in no need to specify the crack front a priori. Namely, the crack front is found as a result of the solution of the boundary value problem. Moreover, the influence of residual stresses developing under the indent due to inelastic compressive behaviour of brittle materials [10,14,15,16,17] is more reliably captured. Some care is needed with respect to the elasticity of the cohesive interface, specifically one should avoid double-counting the elasticity—once in the cohesive law and a second time as part of the bulk behaviour. Nevertheless, the effect of this issue is negligible when cohesive surfaces are only specified along a potential single crack path such as in the case of indentation cracking, or if the stiffness of cohesive surfaces is infinite [18]. Currently, there is an increasing effort to combine cohesive zone models with extended finite element method (XFEM) to model crack growth [19,20,21]. XFEM can avoid remeshing near the crack tip as the crack grows and all other difficulties connected with it. With respect to indentation crack modelling, remeshing is not needed as the indentation cracks extend only over short distances without kinking and a zone of the potential crack formation is covered with cohesive elements. Thus, the application of XFEM does not seem to bring any other benefits in this context. To interpret the results of simulation of the growth of indentation cracks in terms of the linear fracture mechanics, the cohesive (bridging) zone must be significantly smaller than the crack. Hence, great care is needed in applying the simulation results to short crack problems under indentation tests [22,23].

A direct application of the former macroscopic cohesive laws to cleavage fracture, which entails a simple separation of the atomic planes, is not easily workable. Consider (110) cleavage planes in Si crystal. Their interplanar spacing *d* is 1.92 Å. The (110) cracks with [$\bar{1}10$] crack front in Si crystal were analysed using ab initio and gradient elasticity theory in our study [24]. It was shown that the critical crack opening *δ_c_* (interplanar separation) leading to the loss of the crystal bearing capacity is 0.2 nm, the corresponding peak stress is of the order of theoretical strength, and the cohesive energy density 2$\Gamma ≅5.2\;J/m^{2}$. Moreover, the length of the cohesive zone is very small, approximately 0.6 nm. It means that macroscopic FE simulation would require extremely fine mesh, which is often unfeasible. Nguyen and Ortiz [25] suggested a way to the macroscopic form of the cohesive law by considering the cooperative behaviour of a large number *N* of interatomic planes forming a cohesive layer. The thickness of the cohesive layer in FE simulations is given by the local element size *D*. Thus, the number of atomic planes in the cohesive layer is N=2D/d, where the factor 2 was added due to symmetry. Nguyen and Ortiz showed that for sufficiently large *N* the macroscopic critical opening displacement ∆*_c_* and the corresponding macroscopic cohesive stress $\sigma_{\max}\;$for the separation of a single atomic plane asymptotically scale as

(1) $$\Delta_{c}=2\sqrt{\frac{\Gamma N}{C}},\;\;\sigma_{\max}=2\sqrt{\frac{\Gamma C}{N}},$$

where the interplanar modulus *C* depends on a specific material. For the interplanar cohesive potential suggested in [26]

(2) $$\;\phi \left(\delta \right)=2\Gamma -C\delta_{c}\left(\delta +\delta_{c}\right)e^{-\delta /\delta_{c}},$$

the interplanar modulus *C* for (110) planes in Si crystal is

(3) $$C=\frac{2\Gamma }{\delta_{c}^{2}}\;≅1.3\times 10^{20}\;J/m^{4}.$$

For the element size *D =* 0.25 μm, Equation (1) provides $\Delta_{c}≅14.4\;\mathrm{nm}$. While the critical opening displacement ∆*_c_* and the corresponding macroscopic cohesive stress $\sigma_{\max}$ do depend on the element size, the cohesive energy density 2*Γ* is independent of the size element. The aim of this study is to use experimental microindentation data, FE simulations with cohesive zone modelling, and an optimisation procedure to determine the cohesive energy density of single crystals without having to check whether the size of the cohesion zone is considerably less than the crack size and thus to analyse the problems of short cracks. Obviously, such a procedure is particularly suitable for determining fracture properties of MEMS/NEMS parts or thin films using micro/nano indentation tests.

## 2. Materials and Methods

As-received Si crystals (100) with dimensions 50 mm × 50 mm × 3 mm were covered by poly(methyl methacrylate) PMMA before cutting and were precut to 1 cm × 1 cm samples by laser dicer. The cover layer PMMA was removed by acetone, isopropanol and deionised water in an ultrasound cleaner. The final cleaning step was etching of organic residues by oxygen plasma in Diener Plasma cleaner (Diener electronic GmbH, Ebhausen, Germany). Substrates were covered by a double layer of optics resists by spin-coating process. This double layer of resists is important for the lift-off process. The bottom resist was AR-BR 5460 (Allresist GmbH, Strausberg, Germany) and the top resist was AR-P 3540 (Allresist GmbH, Strausberg, Germany). The bottom resist is more sensitive than top resist and this combination of resists creates an undercut in resist layer. The exposure of the resist was done by UV Direct Write Laser system 66+ from Heidelberg Instruments (Heidelberg, Germany). After exposure, samples were developed by AR 300-47 (mixed with deionised water in the ratio 1:1, Allresist GmbH, Strausberg) for 60 s. The residues after developing were removed by oxygen plasma by reactive ion etching in Oxford Instruments Plasma Technology PlasmaPro NGP 80 (Oxford, UK). Optionally, the native oxide layer could be removed by buffered hydrofluoric acid (BOE 7:1 − HF:NH_4_F = 12.5:87.5%).

**Figure 1:**
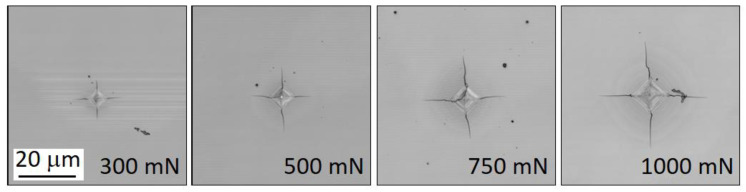
Photographs of indentation with radial cracks.

The indentation tests were load-controlled and performed using Fischerscope H100 XYp equipment (Riley Industries Ltd., Aldridge, UK) with maximal applied force to load cell of 1000 mN acting on standard Vickers diamond indenter with the centreline-to-face angle *ψ* = 68° which was aligned along the cleavage plane {101} in the direction <100> of Si crystal. The minimal applied force, which the equipment can detect, is 0.4 mN with the force resolution 20 μN and depth resolution ±2nm. In this study, the test forces of 300 mN, 500 mN, 750 mN, and 1000 mN, respectively, were applied. Loading stage lasted 20 s followed by 5 s creep and with 20 s long unloading stage. The unloading stage was followed by 5-s long period of constant loading of 0.4 mN. For each of applied forces 25 indentation tests were performed to minimise the experimental error. The crack length was measured by confocal laser microscope Olympus LEXT4000 (Olympus Corporation) from the centre of indentation. Figure 1 shows details of indentation with radial cracks after indentation tests. During indentation the crack behaviour is impossible to optically track because cracks spread under the surface, and they are thus invisible. The first visible radial cracks on the crystal top surface occur during the unloading stage of indentation tests.

**Figure 2:**
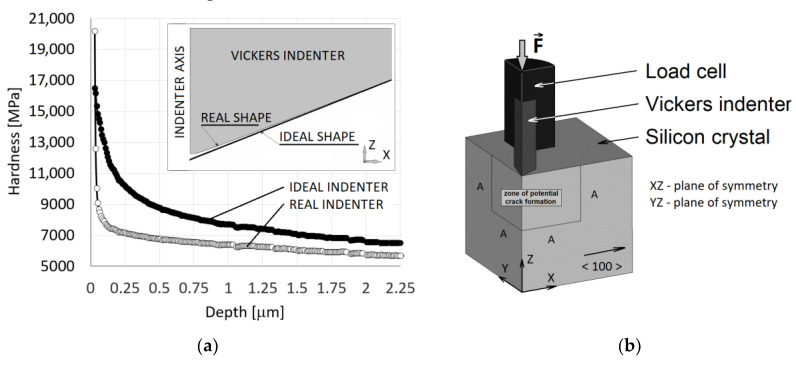
(**a**) FE model of Vickers indentation test on silicon crystal. (**b**) Calibration of the tip shape based on the hardness.

FE model for numerical simulation of the indentation test of Si crystal consisted of a cube with edge length of 200 μm (Si crystal), Vickers diamond indenter and the load cell, see Figure 2a.

The discretisation of the FE model was performed by linear solid elements (SOLID185 in ANSYS software (Release 19.2)) and the contact with friction coefficient *f* = 0.05 between the Si crystal sample and Vickers indenter was defined. The sample was loaded through pushing the indenter into the sample and the indentation depth was gradually increased. As a result, the indenter was deformed, and the loading force was subsequently obtained as a reaction. Two planes of symmetry (XZ, YZ) and the plane XY with prescribed boundary condition (*U_Z_* = 0) representing the crystal storage were used in numerical simulations. Non-elastic response of the Si crystal (denoted as SC), that tends to accommodate the contact stresses under the indenter, was modelled in terms of ideally elastoplastic material defined by Young’s modulus *E_SC_* =129.5 GPa, Poisson’s ratio *ν_SC_* = 0.278, the shear modulus *G_SC_* =79.6 GPa and the yield stress *σ_y_*_, *SC*_ which is initially unknown. In this context it should be noted that the elastic-perfectly plastic material behaviour according to the von Mises yield condition accurately describes the compressive behaviour of many brittle materials [27,28]. Vickers diamond indenter (VDI) was considered as a linear isotropic body defined by Young´s modulus *E_VDI_* = 1220 GPa and Poisson´s ratio *ν_VDI_* = 0.20. Elastic properties of individual components (crystal, indenter) were chosen on the basis of available literature data. Linear isotropic behaviour was also assumed for the load cell (LC) defined by Poisson´s ratio *ν_LC_* = 0.3 and Young´s modulus *E_LC_* which takes the stiffness of the test equipment into account and is also initially unknown. Reduced Young´s modulus *E_r_* is then given by

(4) $$\frac{1}{E_{r}}=\frac{1-\nu_{SC}^{2}}{E_{SC}}+\frac{1-\nu_{VDI}^{2}}{E_{VDI}}+\frac{1-\nu_{LC}^{2}}{E_{LC}}.$$

**Figure 3:**
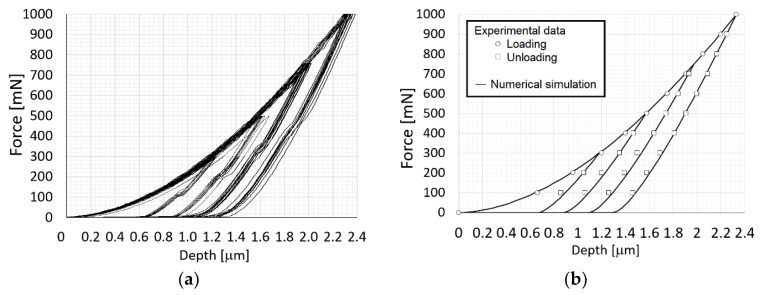
Representative force–depth curves from indentation tests: (**a**) experiment, (**b**) numerical simulation after calibration.

In the first step, the reduced Young´s modulus *E_r_* was searched together with the yield stress *σ_k_*_, *SC*_ based on load-depth curves from indentation tests, see Figure 3a. The indenter tip shape deviation from the ideal shape, see Figure 2a, was also taken into account when searching for the yield strength. Crack initiation and growth was not considered in this stage. It should be pointed out that the effect of cracks on the force-depth curve is negligible for lower loading force values. The nonlinear least-squares routine to get the best fit between the given indentation data and the optimised indentation data, produced by FE analysis, was applied to determine the aforementioned parameters. The corresponding objective functional ℱ(c) is given by, see [29,30,31,32,33]

(5) $$ℱ\left(c\right)=\frac{1}{2}\overset{N}{\underset{i=1}{\sum }}{\left[P_{i}^{comp}\left(c\right)-P_{i}^{exp}\right]}^{2}⤑\min,$$

where *c* is the optimisation variable set given above, $P_{i}^{comp}\left(c\right)$ and $P_{i}^{exp}$ are the predicted and experimental loading force, respectively and *i* denotes a position along the force-depth curve. The yield stress *σ_y_*_, *SC*_ was determined from unloading stage of each of the force-depth curves, where linear behaviour exists (approx. to 10% decrease from maximal value of the applied force) in accordance with Oliver and Pharr method [34]. The best fit was obtained with the yield stress *σ_y_*_, *SC*_ = 6.4 GPa, the value which is close to the values applied for silicon in studies [7,8,10]. Further decrease in the applied force cannot be employed for a correct fitting because the FE model does not include pop-out effect which occurs approximately at 50% decrease of the applied force. The real shape of the indenter tip, which is used in numerical simulations, was found on the basis of a calibration curve of differential hardness, which is performed before the measurement itself. It is therefore a matter of finding a match between the calculated and measured dependence of load vs indentation depth. The shape of the indenter tip and at the same time the required yield strength are calibrated here. The calibration was performed using the universal hardness HU which takes elastic and plastic deformations into account and is defined by the following relation

(6) $$\mathrm{HU}=\frac{P_{\max}}{S_{c}\left(h_{\max}\right)},$$

where *P*_max_ denotes the maximal force acting on the ideal Vickers indenter during a particular indentation test, *h*_max_ denotes the corresponding maximal depth of indentation into the Si crystal, and *S_c_* is the contact area between the indenter tip and the Si crystal. The calibration was solved as an inverse problem by using incremental iteration procedure where the universal hardness and the loading force are known, and the contact area is searched. When the contact area is found the shape of indenter tip is modified and the force is incrementally increased. This procedure runs until the maximal loading (here 1000 mN) is reached. Then the calibration procedure is finished. The ideal and real indenter tip shape of Vickers indenter are shown in Figure 2b. The difference between the ideal and the real shape of indenter tip is irrelevant in terms of the force-depth dependence but essential for the development of cracks in the near vicinity of the indenter tip.

For the crack development modelling, a zone of the potential crack formation of the size *A* × *A* with *A* = 50 μm was defined in both symmetry planes, see Figure 2a. The macroscopic cohesive potential can be obtained from Equations (2) and (3) in terms of the macroscopic opening displacement ∆ as

(7) $$\Phi \left(\Delta \right)=2\Gamma -2\Gamma \left(\frac{\Delta }{\Delta_{c}}+1\right)e^{-\Delta /\Delta_{c}},$$

or

(8) $$\Phi \left(\Delta \right)=2\Gamma -\sigma_{\max}\left(\Delta +\Delta_{c}\right)e^{1-\Delta /\Delta_{c}}.$$

The cohesive traction *T*(Δ) then follows from the derivative of the potential as

(9) $$T\left(\Delta \right)=\Phi^{\prime }\left(\Delta \right)=\frac{\sigma_{\max}}{\Delta_{c}}\Delta e^{1-\Delta /\Delta_{c}}=\frac{2\Gamma }{\Delta_{c}^{2}}\Delta e^{-\Delta /\Delta_{c}}.$$

**Figure 4:**
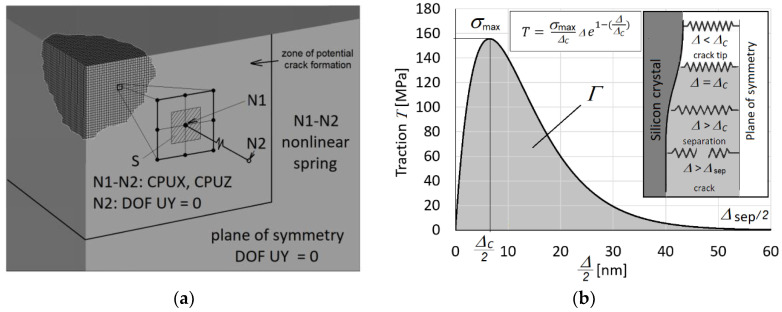
Cohesive zone model (**a**) definition and boundary conditions, (**b**) the traction-crack opening length relationship.

Note that the tangential component was neglected due to the character of loading of potential cracks in Mode I and due to the mechanism of crack formation. It would be possible to extend the model with this feature, however, in our opinion it would not bring a desired benefit. The relationship between the normal traction *T* and crack opening displacement is illustrated in Figure 4b. However, in Figure 4b due to symmetry, only half of the crack opening displacement is displayed. Hence, the area under the traction-half displacement curve is equal to the half of the cohesive energy density 2*Γ*, that is to *Γ*. The cohesive crack zone is realised by means of nonlinear springs in tension. The nonlinear spring response in compression is considered as rigid and the tangential traction is ignored. The crack tip is defined as the point where the crack opening displacement is equal to ∆*_c_* which corresponds to the maximal normal traction *σ*_max_. Numerical simulations were performed for radial cracks propagating along the (101) cleavage plane in the direction [100] and along the (011) cleavage plane in the direction [10], see Figure 2a. The complete elastic–plastic stress field during the unloading stage of the indentation is given by a superposition of the elastic contact stress field $\sigma_{}^{m}$ and a residual stress field $\sigma_{\;}^{r}$ generated due to the permanent deformation $\varepsilon_{}^{p}\;$under the contact. While with decreasing contact force *P*(*t*) the elastic contact stress field decreases, the residual stress field remains largely unchanged and promotes cracks extension. The boundary value problem to be solved during the unloading stage is to find the complete stress-strain field $\sigma =\sigma^{m}+\sigma^{r}$, $\varepsilon =\varepsilon^{m}+\varepsilon^{r}$:

(10) $$\sigma \cdot n=p\left(t\right)\;\;\mathrm{on}\;S_{C}\left(t\right),\;\;n\cdot \sigma \cdot n=T\left(\Delta \right)\;\mathrm{on}\;S_{crack}\left(t\right),$$

(11) $$u^{m}+u^{r}=u=u^{0}\;\;\mathrm{on}\;S_{u},$$

(12) $$\sigma =C:\left(\varepsilon -\varepsilon^{p}\right),\;\;\varepsilon =D:\sigma +\varepsilon^{p}\;\mathrm{and}\;\nabla \cdot \sigma =0\;\mathrm{in}\;\Omega ,$$

where ε satisfies compatibility, $S_{C}\left(t\right)$ denotes the actual contact surface with actual tractions p(t), $S_{crack}\left(t\right)$ is the actual crack surface, $S_{u}$ is the part of the boundary where displacements are prescribed, C and D are the stiffness and compliance tensors respectively, n is a unit normal to the surface. The actual tractions p(t) are related to the actual resultant contact force *P*(*t*) by

(13) $$P\left(t\right)=\int_{S_{C}\left(t\right)}^{}n\cdot p\left(t\right)dS.$$

The total energy *Є* can be expressed in terms of mechanical and residual fields and the cohesive potential Φ(Δ) as

(14) $$\text{Є}=\frac{1}{2}\int_{\Omega }^{}\sigma :\left(\varepsilon -\varepsilon^{p}\right)d\Omega -\int_{S_{C}}^{}p\cdot \left(u^{m}+u^{r}\right)dS+\int_{S_{crack}}^{}\Phi \left(\Delta \right)dS=\;\frac{1}{2}\int_{\Omega }^{}\left(\varepsilon -\varepsilon^{p}\right):C:\left(\varepsilon -\varepsilon^{p}\right)d\Omega -\int_{S_{C}}^{}p\cdot \left(u^{m}+u^{r}\right)dS+\int_{S_{crack}}^{}\Phi \left(\Delta \right)dS.$$

Necessary first-order stationarity condition for the minimisation of the total energy reads

(15) $$\int_{\Omega }^{}C:\left(\varepsilon -\varepsilon^{p}\right):\delta \varepsilon d\Omega -\int_{S_{C}}^{}p\cdot \delta \left(u^{m}+u^{r}\right)dS+\int_{S_{crack}}^{}\frac{2\Gamma }{\Delta_{c}^{2}}e^{-\Delta /\Delta_{c}}\delta \Delta dS+\Phi \left(\Delta_{c}\right)\delta S_{crack}=0.$$

In case of full unloading $u^{m}=\varepsilon^{m}=p=0$ Equation (15) reduces to:

(16) $$\int_{\Omega }^{}C:\left(\varepsilon^{r}-\varepsilon^{p}\right):\delta \varepsilon^{r}d\Omega +\int_{S_{crack}}^{}\frac{2\Gamma }{\Delta_{c}^{2}}e^{-\Delta /\Delta_{c}}\delta \Delta dS+\Phi \left(\Delta_{c}\right)\delta S_{crack}=0,$$

where $\Phi \left(\Delta_{c}\right)=2\Gamma \left(1-2e^{-1}\right)$. Here, it should be emphasised again that the crack tip is defined as the point where the crack opening Δ is equal to $\Delta_{c}$ which corresponds to the maximal normal traction *σ*_max_. The virtual crack area increment $\delta S_{crack}$ is given by

(17) $$\delta S_{crack}=\int_{\partial S_{crack}}^{}\upsilon \cdot \delta Lds,$$

where $\partial S_{crack}$ is the crack front, υ is the local unit normal vector to the crack front and δL denotes the local virtual crack extension. Observe that $\varepsilon^{p}$ and consequently also the residual strain field $\varepsilon^{r}$ depend on the maximal loading force *P*_max._

The identification of the material parameters *Γ* and ∆*_c_* is based upon the best fit between the visible crack length on the top surface and its numerical prediction obtained by FE analysis under full unloading. The optimisation model is

(18) $$J\left(\Gamma ,\Delta_{c}\right)=\overset{M}{\underset{i=1}{\sum }}{\left[L_{ij}^{exp}-L_{j}^{pred}\left(\Gamma ,\Delta_{c}\right)\right]}^{2}\cdot \min,$$

where $L_{ij}^{exp}$ is a measured crack length on the top surface at i-th test, $L_{j}^{pred}\left(\Gamma ,\Delta_{c}\right)$ is its theoretical counterpart, the subscript *j* denotes j-th value of the maximal loading force *P*_max,*j*_ and *M* = 25 is the number of performed indentation tests for each loading force. Simultaneously, the minimisation of the total energy is controlled. Observe, that as the independent cohesive material parameters also *σ*_max_ and ∆*_c_* can be chosen, see Equation (9).

## 3. Results

**Figure 5:**
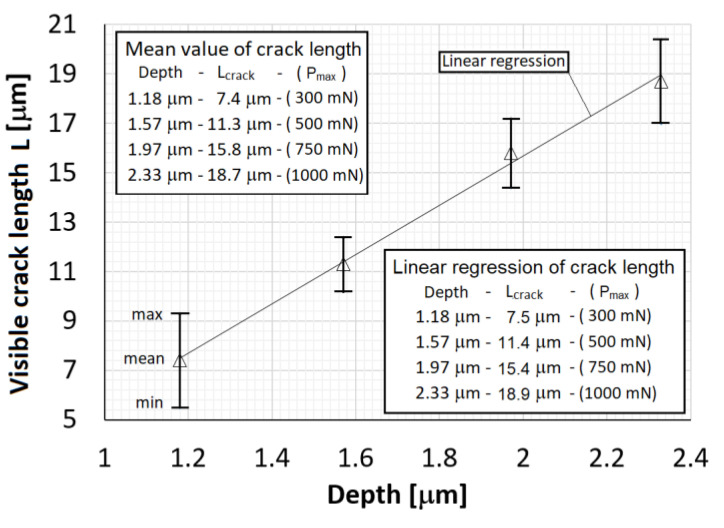
Experimental values of radial crack length.

This section is devoted to the evolution of crack front during indentation tests and determination of the cohesive material parameters using the FE analysis, experimental data and the optimisation model described above. Experimental values of the radial crack length measured on the top surface are shown in Figure 5 for several values of indentation depth and the corresponding maximal loading force. Moreover, linear regression of the experimental data is included revealing that within the applied loading range (up to 1 N) the crack length linearly depends on the indentation depth which corresponds to the maximal loading force *P*_max_. These data are used in the following subsection to find the cohesive energy density of the silicon crystal.

### 3.1. Cohesive Energy Density of Silicon Crystal

The cohesive energy density was obtained from numerical simulations of the indentation cracking and the optimisation model. All the numerical simulations were treated as a direct problem and the radial crack length was one of output parameters. In all simulations the same element size of 0.25 μm was used. As already mentioned the crack length depends significantly on the cohesive energy density 2*Γ* which corresponds to the area under the curve representing the traction-displacement relationship and on the critical opening displacement ∆*_c_*. Both, the cohesive energy density 2*Γ* and the critical opening displacement ∆*_c_* form the output of the inverse problem. The inverse analysis starts with an initial estimate of *Γ*. Subsequently ∆*_c_* is sought so that the total energy *Є* reaches a minimum. In the next step the cohesive energy density 2*Γ* is adjusted to minimise the discrepancy between the measured crack length on the top surface, $L_{ij}^{exp}$, and its theoretical prediction $L_{j}^{pred}\left(\Gamma ,\Delta_{c}\right)$. With a new value of *Γ* a corrected value of ∆*_c_* is sought. This process is iteratively repeated until convergence criteria are met.

**Figure 6:**
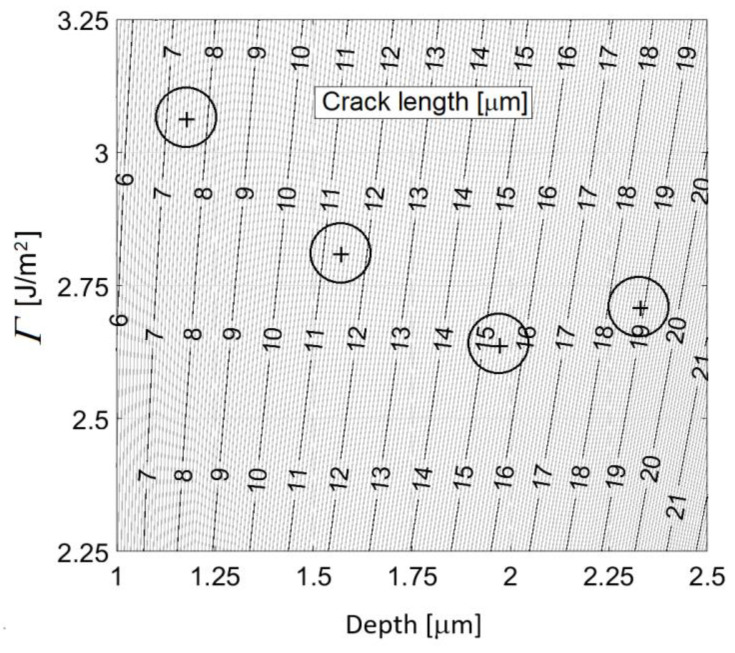
Crack length as function of the indentation depth and the cohesive energy density.

The above procedure was applied for all values of the maximal loading force *P*_max_ = 300 mN, 500 mN, 750 mN, and 1000 mN, and for each of 25 performed indentation tests corresponding to a particular value of *P*_max_. Subsequently, by averaging the iteratively received values of *Γ*, an estimate for the cohesive energy density of the analysed silicon crystal was obtained. The reliability of the used numerical model follows from the comparison of the determined values of *Γ* with values reported in literature. Figure 6 shows the dependency of crack length on the indentation depth and the iteratively received values of *Γ* for particular loading force. If a particular crack length for an appropriate indentation depth is selected in Figure 6, a corresponding value of the cohesive energy density 2*Γ* can be read off. For tested forces/depths, see Figure 5, we get *Γ* = 3.06 J/m^2^ (crack length 7.5 μm and the indentation depth 1.18 μm), *Γ* = 2.81 J/m^2^ (crack length 11.4 μm and indentation depth 1.57 μm), *Γ* = 2.63 J/m^2^ (crack length 15.4 μm and indentation depth 1.97 μm) and *Γ* = 2.70 J/m^2^ (crack length 18.9 μm and indentation depth 2.33 μm). It is seen that with increasing indentation depth the estimate of the cohesive energy density converges to the value 2*Γ* = 5.30 J/m^2^ which agrees well with the silicon cohesive energy density values reported in literature. This convergence is due to a decrease in the measurement error with increasing indentation depth. In general, measurements at a lower indentation depth (a lower applied force) are subject to a larger error. The critical crack opening displacement is $\Delta_{c}=$ 13 nm. Let us however point out again that the critical opening displacement ∆*_c_* does depend on the element size *D*, c.f. Equation (1), which, as already mentioned, is 0.25 μm.

### 3.2. Crack Extension during Indentation Test

**Figure 7:**
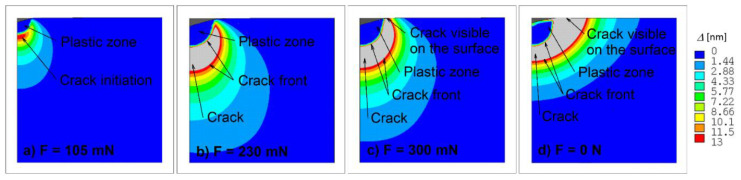
Crack extension during the indentation test for maximal loading force of 300 mN: loading force (**a**) F = 105 mN, (**b**) F = 230 mN, (**c**) F = 300 mN and (**d**) F = 0 N.

With determined parameters $\Gamma ,\Delta_{c}$, crack initiation and growth modelling can be attempted during loading and unloading phases of the indentation test. It is well-known that cracks initiate during the loading phase below the plastic zone which develops under the contact. Further increase of indenter loading leads to crack extension. Figure 7 shows several stages of crack development during the indentation test modelled for maximal loading force of 300 mN.

The instant when this crack occurs can be experimentally captured by monitoring the differential hardness dependence on indentation depth and/or applied force which provides an efficient tool to visualise the indentation induced changes in a tested material such as inception of cracks [35].

**Figure 8:**
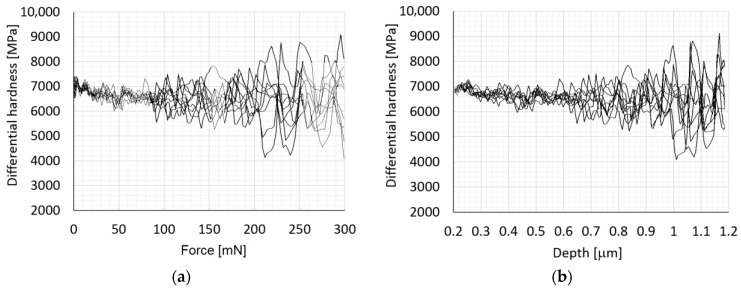
Dependence of differential hardness on (**a**) force, (**b**) depth.

**Figure 9:**
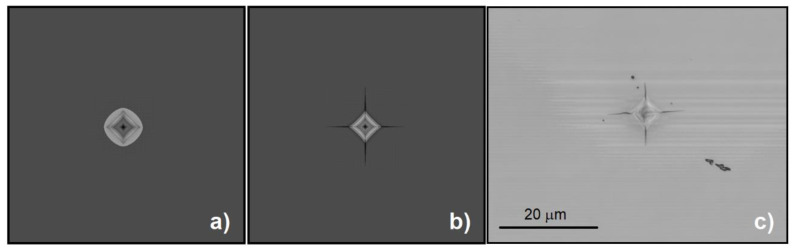
Indentation with the maximal loading force *P*_max_ = 300 mN: (**a**) state at maximal load (numerical simulation), (**b**) state after complete unloading (numerical simulation), (**c**) state after complete unloading (experimental observation).

**Figure 10:**
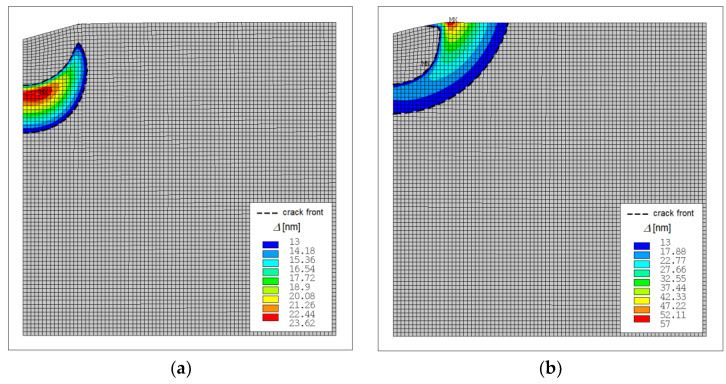
Distribution of the crack opening ∆ along the crack flanks for *P*_max_ = 300 mN (**a**) maximal loading, (**b**) complete unloading.

From measurements, the value of the acting force was approximately 90 mN, see Figure 8a, which is in a good accordance with numerical simulation results (F = 105 mN). Figure 8b presents the same measurements, however the differential hardness is plotted against the recorded indentation depth. One of the advantages of numerical simulations is the visualisation of the invisible crack inception and crack extension. During the unloading stage of indentation test, the originally invisible crack grows to the top surface of the specimen and becomes visible when the applied force decreases from 300 mN to 230 mN, see Figure 7. With further force decrease the crack grows in the radial direction and after complete indenter unloading one can observe cohesive behaviour near the crack tip manifested by cups-like closure, see Figure 9. It is a matter of interest to display numerically predicted distribution of the crack opening along the crack flanks. The results computed for the indentation test with *P*_max_ = 750 mN are displayed in Figure 10. Figure 10 shows the distribution of crack opening along the crack flanks within the cohesive area which allows to identify the crack front. It is clearly seen that crack grows during the unloading stage of indentation test due to residual stress field.

### 3.3. Mesh Density of Cohesive Zone Area

**Figure 11:**
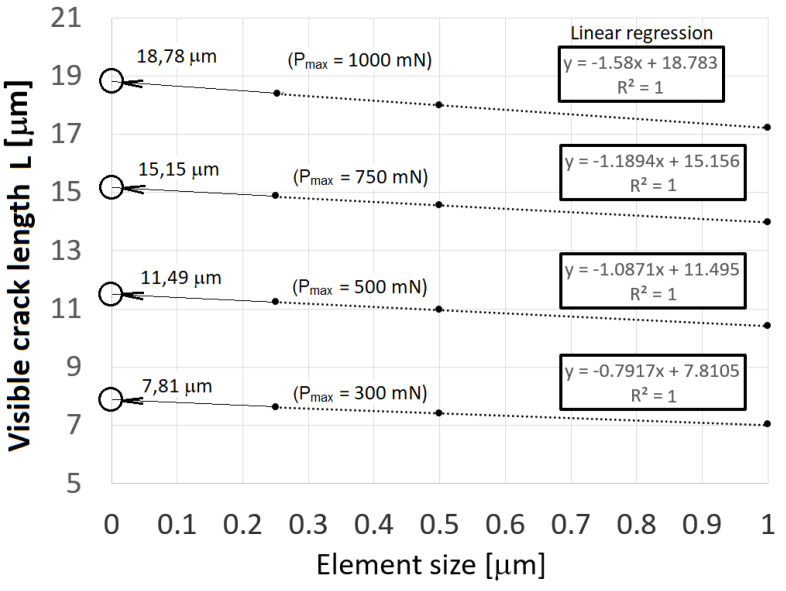
Size effect of mesh density on crack length.

It was already pointed out that the critical opening displacement ∆*_c_* depends on the element size *D.* As mentioned in Introduction, Nguyen and Ortiz [25] suggested a way to the macroscopic form of the cohesive law by considering the cooperative behaviour of a large number *N* of interatomic planes forming a cohesive layer. This approach then shows that ∆*_c_* scales with $\sqrt{D}$. A distinctively weaker dependency on the element size *D* used for discretisation of the cohesive zone area, see Figure 2, was observed for the radial crack length and for the work of cohesive forces as well. Several loading forces were tested and for each the size of the cohesive zone area was adjusted, and thus the discretisation density with respect to the crack surface area. The greater the loading force and, as a result, the greater the crack surface area, the lower is the discretisation error due to the size of the element used. For that reason, the sensitivity to the size of the element (ESIZE) was performed, especially because the ideal size of the element would be at the atomic level—this would of course correspond to a different traction-separation *T*(Δ) dependence. Figure 11 shows a linear regression of the dependency of the crack length on the element size which was used for prediction of the crack length in case when the element size is approaching zero. These data were determined for each indentation loading force and then they were compared with experimental observation and measurement of the radial crack length, see Equation (18).

## 4. Discussion and Conclusions

While previous studies available in the literature, e.g., [7,8,9,10], which use cohesive zone finite element techniques for simulation of indentation cracks in brittle solids, tried to improve methods for the evaluation of material toughness from the indentation load, crack size, hardness, elastic constants, and indenter geometry, this study focuses on the evaluation of the cohesive energy density 2*Γ* from which the material toughness can be easily determined using the well-known Griffith-Irwin formula

(19) $$K_{IC}=\sqrt{\frac{2\Gamma E}{1-\upsilon^{2}}}.$$

With the cohesive energy density determined as 2*Γ* = 5.30 J/m^2^ the Formula (19) gives *K_IC_* = 0.86 MPa·m^1/2^. In contrast to the previous studies, there is no need to control the premise of the linear fracture mechanics that the cohesive zone is much shorter than the crack length. Hence, the developed approach is suitable also for short cracks for which the linear fracture mechanics premise is violated. Besides, in spite of the previous improvements of the indentation cracking formulas, they are still relatively inaccurate to predict the fracture toughness in comparison to the proposed approach based on evaluation of the cohesive energy density.

An integral part of the analysis is modelling of the permanent deformation under the contact since it gives rise to a residual stress field which is the primary driving force for cracks during the unloading process. A reliable model of the permanent deformation and the related residual stress/strain field requires knowledge of the yield stress, the reduced Young´s modulus of the whole system consisting of the load cell, Vickers indenter and the silicon crystal, and also the real shape of the indenter. All these parameters were found using an optimisation procedure which provides the best fit between the experimental indentation data and the optimised indentation data, produced by FE analysis. Specifically, optimal value of the yield strength *σ_y_*_, *SC*_ was 6.4 GPa. The computed residual stress/strain field enters the analysis of the inverse problem for identification of the cohesive energy density 2*Γ* and the critical crack opening displacement ∆*_c_*. The inverse problem solution requires to find the best fit between the visible crack length on the top surface of the silicon crystal and its numerical prediction obtained by FE analysis under full unloading and simultaneously to ensure minimisation of the total energy. The solution results are presented in the form of a diagram which links together the cohesive energy density, the crack length, and the indentation depth, from which the cohesive energy density 2*Γ* can be easily read off for particular crack length and indentation depth. Nevertheless, in case of a lower indentation depth the measurements are subject to a larger error which is reflected in the estimation error of the cohesive energy density. As the indentation depth increases, the error decreases and the estimate of the cohesive energy density converges to the value 2*Γ* = 5.30 J/m^2^.

There are several conflicts concerning the selection of the cohesive interface parameters $\sigma_{\max},\;\Delta_{c}$ in FE modelling of cleavage fracture including the indentation cracks in brittle materials using the cohesive interface model. As already mentioned in Introduction, the ab initio calculations show that the crystal loses its bearing capacity after an interplanar separation of only a few Angstroms. Simultaneously, the peak stresses within the interplanar separation zone are of the order of theoretical strength of crystal. To reach such values in macroscopic FE simulation, extremely fine mesh would be required and full atomistic resolution in the vicinity of the crack would be necessitated, which is however unfeasible and impractical. Therefore, a suitable transformation of atomistic binding relation leading to macroscopic cohesive law is needed. A way to the macroscopic form of the cohesive law was suggested by Nguyen and Ortiz [25] as mentioned in Introduction. As a result, the cohesive interface parameters $\sigma_{\max},\;\Delta_{c}$ in any macroscopic cohesive law for the cleavage fracture differ by orders from their physical atomistic counterparts, however with the cohesive energy density (critical fracture energy) 2*Γ* remaining unchanged. There are other limitations for the choice of the parameter $\sigma_{\max}$ which were thoroughly investigated and discussed in [7]. It was shown that the parameter $\sigma_{\max}$ should be chosen to be lower than ≈ 0.2 *σ_y_* to ensure initiation of crack in a linear fracture mechanics context. Moreover, care is needed when changing $\sigma_{\max}$ because then the crack bridging zone also changes which affects the choice of cohesive element size *D*. In the papers [7,10] $\sigma_{\max}$ was chosen from the range <0.5,1> GPa, the typical value of the yield strength was $\sigma_{y}=5\;\mathrm{GPa}$, and the typical value of the Young modulus was *E* = 200 GPa. The fracture toughness *K_IC_* ranged from 0.7 to 1 MPa.m^1/2^. All these papers used the bilinear form of the cohesive traction—separation law. In this paper, the parameter $\sigma_{\max}$, and/or the critical opening displacement ∆*_c_* together with the cohesive energy density 2*Γ* were selected to provide best fit between the visible crack length on the top surface and its numerical prediction according to Equation (18). The optimal values were found as $\sigma_{\max}=150\;\mathrm{MPa}$, ∆*_c_* = 13 nm, and 2*Γ* = 5.30 J/m^2^. It is interesting to notice that the asymptotic scaling rule in Equation (1)_1_ predicts the macroscopic critical opening displacement as ∆*_c_* ≅ 14.4 nm, see the text below Equation (3).

The reliability of the developed model is evidenced by the determined value of the cohesive energy density, which is in a good accordance with the values reported in the literature. Furthermore, the reliability of the model follows from the comparison of numerical simulation results with the measured differential hardness data which provide an estimate of the loading force for the indentation induced cracks inception during the loading stage. The proposed approach also seems appropriate for toughness evaluation of hard coatings bonded to a brittle substrate. Studies along this line will be left for a future work.

## Data Availability

Data is contained within the article.

## Nomenclature

*c*	optimisation variable
*d*	interplanar spacing
*h*_max_	maximal depth of indentation
*f*	friction coefficient
*i*	position along the force-depth curve
p	actual tractions
n	unit normal to the surface
*A*	size of cohesive zone
*C*	interplanar modulus
*D*	finite element size
C	stiffness tensor
D	compliance tensor
ℱ	objective functional
*HU*	universal hardness
*N*	number of interatomic planes forming a cohesive layer
*E_LC_*	Young´s modulus of test equipment load cell
*E_r_*	Reduced Young´s modulus
*E_SC_*	Young´s modulus of the Si crystal
*E_VDI_*	Young´s modulus of Vickers diamond indenter
*G_SC_*	shear modulus of the Si crystal
$K_{IC}$	material fracture toughness
$L_{ij}^{exp}$	measured crack length on the top surface at i-th test
$L_{j}^{pred}$	predicted crack length
*P*	contact force
$P_{}^{comp}$	predicted loading force
$P_{}^{exp}$	experimental loading force
*P*_max_	maximal force acting on the ideal Vickers indenter
*S*_c_	contact area between the indenter tip and the Si crystal
$S_{crack}$	crack surface
$S_{u}$	part of the boundary where displacement are prescribed
*T*	cohesive traction
$\varepsilon_{}^{p}$	permanent deformation
*δ*	microscopic crack opening
*δ_c_*	critical microscopic crack opening (interplanar separation)
δL	local virtual crack extension
υ	local unit normal vector to the crack front
***ν*** *_SC_*	Poisson´s ratio of the Si crystal
*ν* *_VDI_*	Poisson´s ratio of Vickers diamond indenter
*ν* *_LC_*	Poisson´s ratio of test equipment load cell
*ψ*	centreline-to-face angle
*Є*	total energy
σ	complete stress-strain field
$\sigma_{}^{m}$	elastic contact stress field
$\sigma_{\;}^{r}$	residual stress field
*σ_y, SC_*	yield stress of the Si crystal
*σ_max_*	peak cohesive traction
2*Γ*	cohesive energy density (critical fracture energy)
∆	macroscopic opening displacement
∆*_c_*	corresponding damage-initiating displacement
∆*_sep_*	failure displacement
ϕ	interplanar cohesive potential
